# F53. DOSE REDUCTION OF HIGH-DOSE FIRST-GENERATION ANTIPSYCHOTICS OR SWITCH TO ZIPRASIDONE IN LONG-STAY PATIENTS WITH SCHIZOPHRENIA: A 1-YEAR DOUBLE-BLIND RANDOMIZED CLINICAL TRIAL

**DOI:** 10.1093/schbul/sby017.584

**Published:** 2018-04-01

**Authors:** Jan Bogers, Raphael Schulte, Broekman Theo, Lieuwe de Haan

**Affiliations:** 1 MHO Rivierduinen; 2 MHO North Holland North; 3 Bureau Beta; 4 Academic Medical Center, University of Amsterdam

## Abstract

**Background:**

Long-stay patients with severe schizophrenia are frequently treated with high doses of first-generation antipsychotics (FGA). Dose reduction or switching to ziprasidone may reduce the severity of negative symptoms or side effects.

**Methods:**

In a randomized double-blind trial, we compared the effect of FGA dose reduction (to equivalent of 5 mg/day haloperidol) (n=24) or switching to ziprasidone 160 mg/day (n=24). Negative symptoms after 1 year of treatment were primary outcome measure. Treatment failure was defined as a prolonged (>4 weeks) or repeated relapse.

**Results:**

Negative symptoms did not change significantly during dose reduction nor was there a significant difference between treatments. Neurological side effects diminished in both conditions. Positive symptoms, excited symptoms, and emotional distress worsened over time with ziprasidone, resulting in a significant difference in favour of FGA dose reduction. More patients in the ziprasidone condition (46%) than in the FGA condition (21%) relapsed. Although some recovered within 4 weeks, treatment failed in 25% of the patients in the ziprasidone condition and in 17% of the patients in the FGA condition (non-significant differences). In about 80% of patients, doses could be reduced without a prolonged increase in symptom severity.

**Discussion:**

In long-stay patients with severe schizophrenia, reducing high doses of FGA to a dose equivalent of 5 mg/day haloperidol or switching to ziprasidone did not improve negative symptoms. Reducing antipsychotic doses was feasible in most patients, although the risk of relapse is substantial. Neither FGA dose reduction nor ziprasidone seems an adequate alternative to clozapine for treatment-resistant schizophrenia.

